# Development of a transformer-based deep learning algorithm for diabetic peripheral neuropathy classification using corneal confocal microscopy images

**DOI:** 10.3389/fcell.2024.1484329

**Published:** 2024-10-14

**Authors:** Wenqu Chen, Danling Liao, Yuyang Deng, Jianzhang Hu

**Affiliations:** Department of Ophthalmology, Fujian Medical University Union Hospital, Fu Zhou, China

**Keywords:** confocal microscopy, convolutional neural network, diabetic neuropathy, Swin transformer network, deep learning

## Abstract

**Background:**

Diabetic peripheral neuropathy (DPN) is common and can go unnoticed until it is firmly developed. This study aims to establish a transformer-based deep learning algorithm (DLA) to classify corneal confocal microscopy (CCM) images, identifying DPN in diabetic patients.

**Methods:**

Our classification model differs from traditional convolutional neural networks (CNNs) using a Swin transformer network with a hierarchical architecture backbone. Participants included those with (DPN+, n = 57) or without (DPN−, n = 37) DPN as determined by the updated Toronto consensus criteria. The CCM image dataset (consisting of 570 DPN+ and 370 DPN− images, with five images selected from each participant’s left and right eyes) was randomly divided into training, validation, and test subsets at a 7:1:2 ratio, considering individual participants. The effectiveness of the algorithm was assessed using diagnostic accuracy measures, such as sensitivity, specificity, and accuracy, in conjunction with Grad-CAM visualization techniques to interpret the model’s decisions.

**Results:**

In the DPN + group (n = 12), the transformer model successfully predicted all participants, while in the DPN− group (n = 7), one participant was misclassified as DPN+, with an area under the curve (AUC) of 0.9405 (95% CI 0.8166, 1.0000). Among the DPN + images (n = 120), 117 were correctly classified, and among the DPN− images (n = 70), 49 were correctly classified, with an AUC of 0.8996 (95% CI 0.8502, 0.9491). For single-image predictions, the transformer model achieved a superior AUC relative to the ResNet50 model (0.8761, 95% CI 0.8155, 0.9366), the Inception_v3 model (0.8802, 95% CI 0.8231, 0.9374), and the DenseNet121 model (0.8965, 95% CI 0.8438, 0.9491).

**Conclusion:**

Transformer-based networks outperform CNN-based networks in rapid binary DPN classification. Transformer-based DLAs have clinical DPN screening potential.

## Introduction

Diabetic peripheral neuropathy (DPN), which affects more than half of people with diabetes, is the most common complication of this disease (Dyck et al., 1999; Pop-Busui et al., 2017). However, the subtle signs of this condition can go unnoticed until it is firmly developed and irreversible (Dyck et al., 2011). Early detection of diabetic neuropathy is vital to halt its progression and reduce the associated morbidity and mortality risks (Alam et al., 2017; Iqbal et al., 2018). Presently, the primary method for screening for DPN is the 10 g monofilament test. The 10 g monofilament test involves applying a standardized nylon monofilament to various points on the foot to assess the patient’s ability to feel pressure, serving as a simple screening tool for detecting loss of protective sensation (Kumar et al., 1991). However, this method relies on the subjective response of patients and is limited in its effectiveness in detecting small fibers initially affected by DPN (Dyck and Giannini, 1996; Richard et al., 2014; Ziegler et al., 2014). Evaluating small-fiber function often requires an invasive skin biopsy (Lauria et al., 2009). Straightforward indicators for identifying DPN early are lacking in clinical practice (Selvarajah et al., 2019).

Growing evidence supports the value of corneal confocal microscopy (CCM) in detecting DPN. CCM offers a swift, noninvasive method to precisely and objectively measure changes in the corneal subbasal nerve plexus in diabetic patients (Chen et al., 2015; Liu Y.-C. et al., 2021). In DPN, CCM reveals significant alterations in nerve fibers, including a reduction in nerve fiber density, changes in morphology characterized by thinner and more tortuous fibers, and a loss of nerve branching (Yu et al., 2022). These changes collectively indicate nerve damage and degeneration, serving as critical markers for the assessment of neuropathy severity. It is highly reproducible and can detect small fiber function (Devigili et al., 2019; Li et al., 2019). Moreover, it demonstrates high sensitivity and specificity in quantifying early damage in DPN patients (Petropoulos et al., 2013; 2014; Williams et al., 2020; Roszkowska et al., 2021) and can predict the occurrence of diabetic neuropathy (Pritchard et al., 2015). This approach is a reliable and noninvasive alternative to skin biopsy (Ferdousi et al., 2021; Perkins et al., 2021). However, despite its advantages, the CCM is not currently used for clinical screening for DPN in patients. This is because large prospective studies are needed to confirm the reliability of CCM (Sloan et al., 2021), and it is essential to ensure precise feature extraction from CCM images.

In the past decade, artificial intelligence (AI), primarily deep learning, has become widely used in clinical diabetic retinopathy screening, significantly reducing the demands on screening resources (Rajesh et al., 2023). AI models for diagnosing DPN from CCM images have shown promising results. Dabbah et al. (2011) developed a dual-model system for automated detection that can extract nerve fibers from CCM images. They improved the automated software by employing dual-model properties across multiple scales, achieving performance levels similar to manual annotation (Chen et al., 2017). The model, which lacks convolutional layers, necessitates additional data preprocessing and may lead to overfitting during the training process, thus imposing constraints on its performance. Convolutional neural networks (CNNs), a branch of deep learning, achieve “end-to-end” classification without requiring specific instance parameters (LeCun et al., 2015). It has excelled in the automatic analysis of CCM images. Preston et al. (2022) introduced an approach for classifying peripheral neuropathy that eliminates the need for nerve segmentation, which refers to the process of isolating and identifying individual nerve fibers in images for analysis. Additionally, they integrated attribution techniques to provide transparency and interpretation of the decision-making process. However, efficiently and accurately obtaining corneal nerve features from CCM images remains one of the most challenging issues in the intelligent analysis of CCM images. We have noted that another deep learning algorithm (DLA), the Swin transformer network, has shown strong performance in image classification (Liu Z. et al., 2021). The Swin transformer network is a type of transformer architecture that utilizes a hierarchical design, allowing it to effectively process images at multiple resolutions. Unlike CNNs, the Swin transformer employs a unique shifted window mechanism for self-attention, which enables efficient information capture across different parts of the image while maintaining computational efficiency. However, its application in classifying CCM images has not been reported.

In this study, we used a transformer-based DLA to classify CCM images for detecting DPN. We aimed to confirm the feasibility of the transformer architecture for CCM image classification tasks by comparing it with traditional CNN models.

## Methods

### Participants

Ninety-four participants diagnosed with diabetes mellitus (DM) underwent assessment for the presence (DPN+, n = 57) or absence (DPN−, n = 37) of DPN based on the updated Toronto consensus criteria (Table 1). These criteria require evidence of neuropathy, along with at least one abnormality in two nerve electrophysiology parameters: peripheral nerve amplitude and peripheral nerve conduction velocity (Tesfaye et al., 2010). Participants were recruited from outpatients of Fujian Medical University Union Hospital, Fuzhou, China, 2024/01–2024/06. Before any assessments took place, all participants provided informed and valid consent. Each participant underwent comprehensive neuropathy and CCM evaluation. Those who had experienced neuropathy in the past (excluding diabetes), had current or recurring diabetic foot ulcers, lacked sufficient vitamin B12 or folate, had a history of corneal disease or surgery, or wore contact lenses were not included in the study. The research adhered to the Declaration of Helsinki, and approval from ethical and institutional bodies was secured prior to participants beginning the study. Ethics approval was obtained from the Ethics Committee of Fujian Medical University Union Hospital (NO. 2024KY112).

**TABLE 1:** Demographic and clinical profiles of participants with DM.

Variables	DPN−	DPN+	*P* value
n	37	57	
Age (years)	54.7 ± 12.7	57.7 ± 11.3	0.164
Diabetes duration (years)	7.7 ± 6.5	13.7 ± 7.6	<0.001
HbA1c (%)	8.0 (7.1,10.0)	8.7 (7.6,10.1)	0.186
BMI (kg/m^2^)	23.9 ± 3.4	24.2 ± 3.3	0.716
eGFR (mL min^−1^ L^−1^)	108.4 ± 26.2	105.6 ± 37.8	0.712
NDS	0.0 (0.0, 2.0)	5.0 (3.0, 6.0)	<0.001
SSNA (μV)	12.0 (9.0, 16.0)	5.0 (3.0, 8.0)	<0.001
SSNCV (m/s)	55.0 (53.0, 60.0)	48.0 (39.0, 54.0)	<0.001

BMI, body mass index; HbA1c, Hemoglobin A1c; eGFR, estimated glomerular filtration rate; NDS, neuropathy disability score; SSNA, sural sensory nerve amplitude; SSNCV, sural sensory nerve conduction velocity; Data that follows a normal distribution are presented as the mean ± SD; if not, it is expressed as median (P25, P75).

### Image dataset

The dataset consists of corneal subbasal nerve plexus images of diabetic individuals. The images were obtained at a resolution of 400 × 400 μm with a confocal laser microscope (Heidelberg Engineering, Heidelberg, Germany). Images were acquired from the central cornea at the subbasal nerve plexus using section mode, capturing each image at a resolution of 400 × 400 μm (384 × 384 pixels). The images were exported in JPEG file format. During imaging, participants’ heads were fixed, and they were instructed to gaze straight ahead, ensuring that the laser reflection point remained at the center of the cornea. During data collection, the CCM of each participant yielded a spectrum of 20 to 40 digital images per eye. After a rigorous screening by medical experts (JZH and WQC), which involved excluding images marred by blurriness or captured from regions deemed inappropriate, quintets of images per eye were meticulously selected for analytical purposes. The selection was based on the following principles: first, the images had to be of high quality with good contrast and clearly visible nerves; second, they were selected from the central cornea; and third, we aimed to represent a range of corneal nerve densities, choosing images that showed the lowest to the highest density (Kalteniece et al., 2017). The final dataset consisted of images from individuals diagnosed with DPN (n = 570) and individuals diagnosed without DPN (n = 370).

### Dataset preparation

The dataset was arbitrarily stratified among training, validation, and test subsets following a proportion of 7:1:2, anchored on individual participants. The data were divided according to the original distribution ratio, consisting of 660 for the training set (DPN+ 400, DPN− 260), 90 for the validation set (DPN+ 50, DPN− 40), and 190 for the test set (DPN+ 120, DPN- 70). The training set was utilized for model data training and parameter adjustment, and the validation set was employed to assess the effect in the training phase. The test set data were utilized to assess the final effectiveness of the model.

Before being input into the model, the images underwent preprocessing operations. We resized images from 384 × 384 pixels to 224 × 224 pixels using the bilinear interpolation method to meet the input specifications of the model. We normalized the values to [−1, 1] using a mean of 0.5 and a standard deviation of 0.5 across the three channels during image input to optimize training.

### Network architecture and different backbone

**FIGURE 1:**
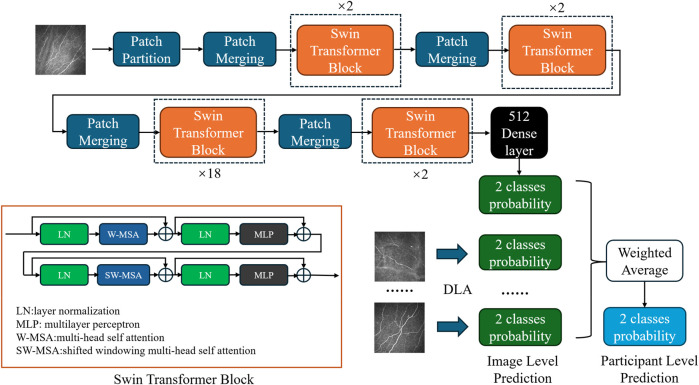
Diagram of the DLA based on the modified Swin transformer network. Each blue block represents patch merging, which is primarily employed to adjust the dimensionality of the input after initial segmentation into patches. Each orange block represents a Swin transformer block, which utilizes a concatenation of multihead self-attention and shifted windowing multihead self-attention mechanisms to facilitate information exchange among small patches. The black block represents the subsequent classifier, which comprises dense and dropout layers, to obtain the final classification probabilities. In the DLA network, a single-image input generates image-level predictions. Probabilities from multiple images belonging to the same participant were weighted averaged to obtain participant-level predictions.

Our classification model employs the Swin transformer network (Liu Z. et al., 2021) as its backbone (Figure 1). Compared to the traditional CNN, the transformer structure introduces a hierarchical architecture that processes features at different resolutions by training the image in small patches. The self-attention computation of the transformer is confined to fixed-size local windows. The Swin transformer leverages a shifted window mechanism, alternating the positions of these windows in adjacent layers, enabling the model to capture information across windows while avoiding the high computational cost of global self-attention. After obtaining 1024-dimensional features extracted by the network, we added a compact stratum with 512 neurons, sequenced by a dropout layer featuring a rate of 0.7. Finally, the features passed through a binary dense layer, and the softmax activation function was used to output the binary classification result. The network continued training on the basis of pretrained model weights from ImageNet1000 (Deng et al., 2009).

In the model configuration for the Swin Transformer, the patch size is set to 4, and the window size is set to 7. The base model has an embedding dimension of 128, with the depth of each Swin Transformer block set to 2, 2, 18, and 2, and the number of heads set to 4, 8, 16, and 32, respectively. For the tiny model, the embedding dimension is set to 96, with the depth of each block configured as 2, 2, 9, and 2, and the number of heads as 3, 6, 12, and 24, respectively. During the model training process, the batch size was set to 32, and the Stochastic Gradient Descent optimizer was employed. The initial learning rate was set to 0.005, and the learning rate scheduler used was CosineAnnealingLR. Cross-entropy loss was chosen as the loss function, and the model was trained for 150 epochs.

We also selected other models for training to compare their effectiveness. ResNet50 (He et al., 2016), DenseNet121 (Huang et al., 2018), and InceptionV3 (Szegedy et al., 2016) were selected as different backbone networks for training. These network structures are all based on the CNN architecture. The experimental training settings remained consistent except for changes in the input size of the individual networks.

The results were generated using a PC featuring an Intel Core i7-13700K processor with 32 GB of RAM and an Nvidia GeForce RTX 4070 Ti. The model was formulated and trained using Python 3.9 and PyTorch 2.0.0.

### Performance evaluation

Each model generated a respective confusion matrix, elucidating the discrepancy between the actual classification of images and the predictions rendered by the model. A suite of standard metrics derived from the confusion matrix for evaluating classification models was computed, encompassing accuracy, sensitivity, specificity, and the F1 score.

Accuracy is determined as the ratio of accurate predictions to the overall number of predictions.

Recall, also called sensitivity, represents the number of predicted positives relative to the total number of actual positives.

Precision is defined as the ratio of true positive instances among all predicted positives, which gauges the impact of false positives.

F1-score is a statistical measure that represents the harmonic mean of precision and recall, balancing the trade-off between these two metrics. It offers a more comprehensive evaluation of model performance compared to using accuracy alone (Sokolova and Lapalme, 2009).

Area Under the Curve (AUC) is a crucial metric for evaluating classification models. It quantifies the model’s ability to distinguish between classes, summarizing the trade-offs between sensitivity and specificity at various thresholds. A higher AUC value indicates better model performance in differentiating between the positive and negative classes, making it an essential indicator of a model’s potential effectiveness in clinical settings.

Grad-CAM technology (Selvaraju et al., 2020) is employed to visualize the areas within images that the model prioritizes for decision-making, offering insights into the model’s interpretability.

Given that five images were taken from each participant from both the left and right eyes, with each image yielding a binary probability output, the evaluation of the model efficacy in discerning the status of participants necessitates a comprehensive approach. Therefore, the probability outputs associated with all images from a single participant are combined through a weighted mean calculation. A patient is defined as DPN + when the weighted mean of DPN+ is greater than that of DPN−, and as DPN− when the weighted average of DPN+ is less than or equal to that of DPN−. The final output serves as the criterion for assessment at the participant level.

## Results

The confusion matrix table generated from the test set using the Swin transformer network as the prediction model for backbone network training is shown in Tables 2, 3. Among the DPN + patients (n = 12), the model successfully predicted all participants. According to the single-image confusion matrix, 117 DPN + images (n = 120) were accurately categorized, and 3 images were inaccurately categorized as DPN−.

**TABLE 2:** Single-image confusion matrix from the Swin transformer network.

True class	Predicted class	
	DPN−	DPN+
DPN-	49	21
DPN+	3	117

**TABLE 3:** Single-participant confusion matrix from the Swin transformer network.

True class	Predicted class	
	DPN-	DPN+
DPN−	6	1
DPN+	0	12

The performance indicators for a single image are shown in Table 4. The network trained with the Swin transformer architecture reached the highest accuracy of 0.8789, in contrast to other classification networks. The best F1 score was 0.9084, and the highest recall was 0.9500. The AUC reached 0.8996 (0.8502, 0.9491). Overall, the networks based on the transformer architecture achieved superior results.

**TABLE 4:** Single-image and single-participant classification indicators.

Classification	ModelName	Acc	Precision	Recall	F1	AUC	95% CI
Single-image	Swin_transformer_base	0.8789	0.8702	0.95	0.9084	0.8996	0.8502, 0.9491
	Swin_transformer_tiny	0.8684	0.8682	0.9333	0.8996	0.8977	0.8483, 0.9472
	DenseNet121	0.8737	0.8871	0.9167	0.9016	0.8965	0.8438, 0.9491
	Inception_v3	0.8632	0.8561	0.9417	0.8968	0.8802	0.8231, 0.9374
	ResNet50	0.8684	0.88	0.9167	0.898	0.8761	0.8155, 0.9366
	ResNet152	0.8579	0.8605	0.925	0.8916	0.8886	0.8354, 0.9419
Single-participant	Swin_transformer_base	0.8947	0.9167	0.9167	0.9167	0.9405	0.8166, 1.0000

**FIGURE 2:**
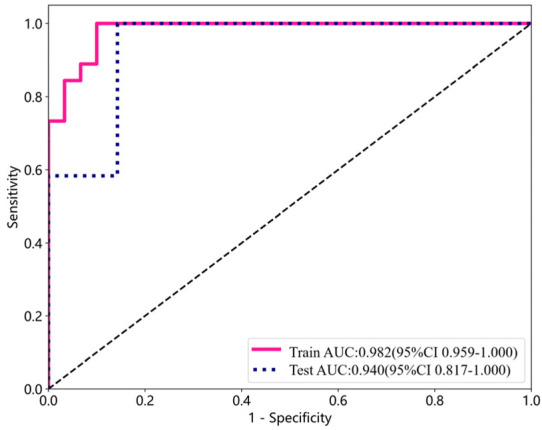
ROC curve analysis for detecting DPN using training and test subsets from a single participant.

The performance indicators for a single participant are shown in Table 4. Apart from the area beneath the ROC curve, all other model performance metrics align with our proposed model. The overall classification accuracy reached 0.8947, with the AUC achieving an even higher value of 0.9405 (0.8166, 1.0000). Figure 2 depicts the AUC performance of our suggested method, delineated separately for the training and test subsets across a single participant.

**FIGURE 3:**
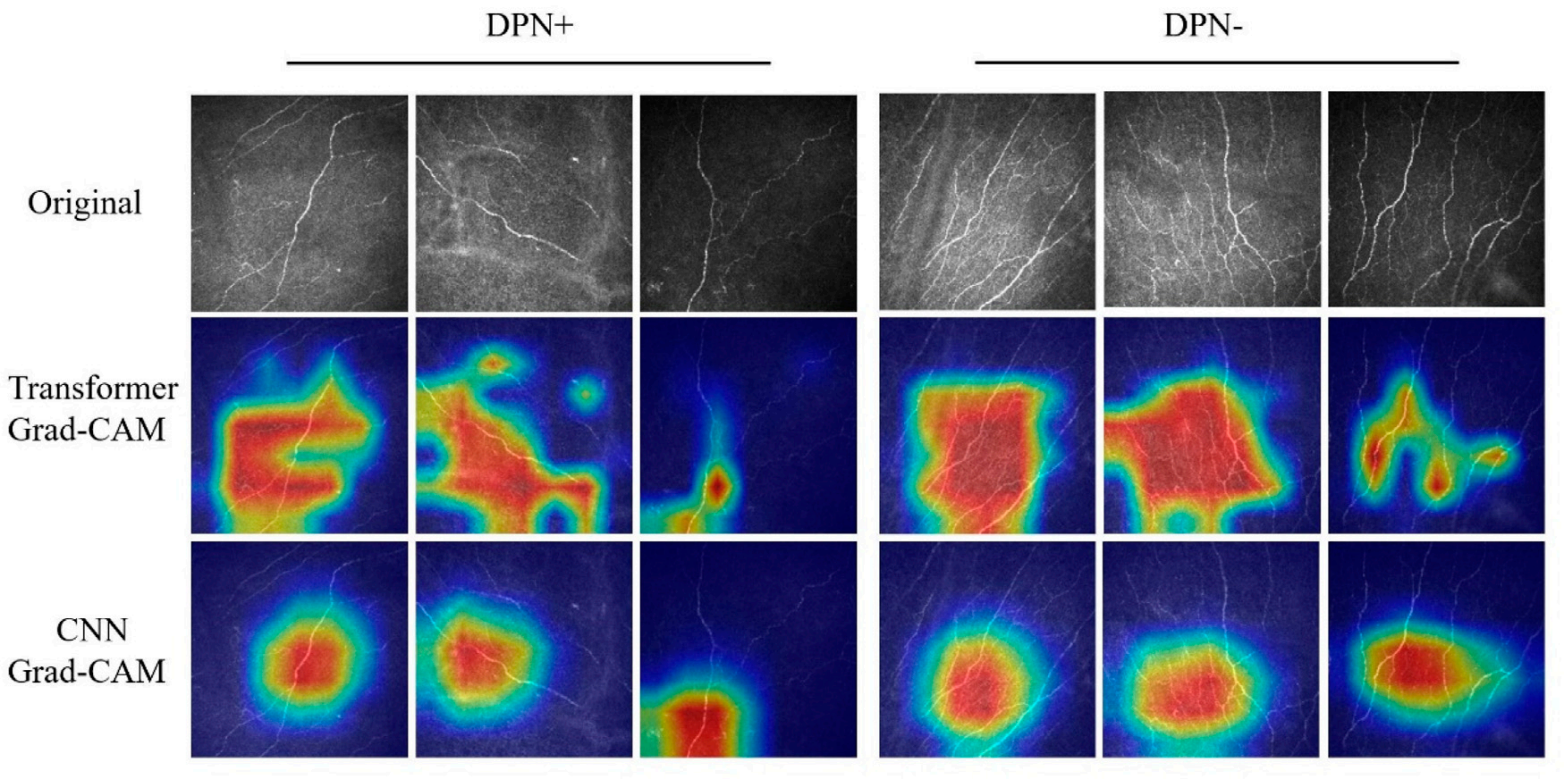
Grad-CAM images of DPN+ (left 3 columns) and DPN− (right 3 columns) patients accurately detected using the transformer and CNN models. Upper section, original images; center section, transformer Grad-CAM images; lower section, CNN Grad-CAM images.

Figure 3 presents the various CCM images within the test set across distinct models, alongside the Grad-CAM images generated for each case. The networks based on the transformer architecture tend to exhibit more refined detail in the regions identified for image judgment during the presentation process than those based on CNN architectures.

## Discussion

In our research, we employed an advanced DLA to analyze CCM images of corneal nerves, simulating a clinical screening environment, to distinguish diabetic patients with DPN from the diabetic population. The algorithm performed well in this binary classification task without requiring nerve segmentation (AUC 0.9405; sensitivity 0.8947; specificity 0.9167), highlighting its clinical utility in accurately identifying DPN patients. Our experiments showed that in classifying DPN using deep learning networks, networks based on transformer architectures outperform those based on CNN architectures.

Current clinical criteria of DPN typically include a combination of clinical history, physical examination, and nerve electrophysiology, but they often fail to capture the nuances of nerve damage early on (Atmaca et al., 2024). Timely detection and assessment of small nerve fiber damage is key to screening for DPN. CCM, as a non-invasive examination method, can provide accurate biomarkers for small nerve fiber damage (Carmichael et al., 2022).

Some studies have utilized AI-based CNN approaches to analyze corneal nerve images to classify DPN patients (Scarpa et al., 2020; Williams et al., 2020; Salahouddin et al., 2021; Preston et al., 2022; Meng et al., 2023). Williams et al. (2020) employed a U-Net CNN architecture for analyzing and quantifying corneal nerves, achieving an AUC of 0.83, specificity of 0.87, and sensitivity of 0.68 in distinguishing DPN + patients. Preston et al. (2022) utilized a ResNet-50-based CNN to classify corneal nerve images for DPN, attaining a recall of 0.83, precision of 1.0, and an F1 score of 0.91. Salahouddin et al. (2021) employed a U-Net-based CNN to distinguish individuals with DPN from those without DPN, demonstrating a sensitivity of 0.92, a specificity of 0.8, and an AUC of 0.95. Meng et al. (2023) utilized a modified ResNet-50 CNN to achieve dichotomous classification between DPN+ and DPN− patients, with a sensitivity of 0.91, a specificity of 0.93, and an AUC of 0.95.

In contrast to previous CNN-based models, our study utilizes the Swin transformer as the backbone network for training. In our research, the models generally demonstrated superior performance, as evidenced by higher AUC values than those trained with traditional architectures. The Grad-CAM visualizations further revealed that Swin transformer-based models excel in identifying nuanced details of neural components, indicating that they extract features with greater precision and depth than conventional CNN architectures. Our approach, which contrasts with analogous binary classification methods, is more advanced and demonstrates enhanced sensitivity. The pronounced recall of the model indicates an enhanced sensitivity toward detecting neurological abnormalities, notwithstanding the possibility of misdiagnosis in certain instances. This heightened sensitivity is pivotal in minimizing the likelihood of overlooking such conditions, thereby rendering the model exceptionally beneficial for preliminary screenings.

Due to the limited coverage of each corneal image, individual images may offer incomplete representations of the overall corneal nerve. Relying solely on a single image might not provide a sufficiently comprehensive depiction for an accurate diagnosis. Scarpa et al. (2020) simulated the clinical decision-making process by utilizing multiple corneal nerve images from a single eye to assess DPN, which supported this standpoint. In our study, aggregating the prediction outcomes from multiple images for classification led to an overall improvement in the composite metrics across all models. This indicates that integrating judgments from multiple images yields more accurate results than relying on the assessment of a single image (Meng et al., 2023). This approach corresponds more with the judgments made in practical scenarios. Clinicians often find it difficult to make assessments based on a single image. This approach both improves the precision of model predictions and mirrors the nuanced decision-making process in clinical settings.

However, the sample size in our study, particularly for the DPN- group, is limited. A larger cohort would improve statistical power and generalizability. In addition, our model’s performance needs to be validated on external datasets to ensure its reliability. Given that data collection is a challenging process, we are concurrently gathering data from multiple centers.

Our DLA-based DPN screening method showed superior performance compared to the currently used monofilament tests (Wang et al., 2017), which demonstrated a sensitivity of 0.53 and specificity of 0.88. Despite the limitations of a smaller dataset, our study still attained a reasonable level of classification accuracy. There are currently no reports of AI-based CCM deployed in real-world settings for screening DPN. Based on previous experience, previous studies on diabetic retinopathy have suggested that AI performs less effectively in clinical practice than in laboratory validation (Kanagasingam et al., 2018). Therefore, large-scale prospective clinical studies are crucial for AI-based DPN screening.

## Conclusion

The transformer-based networks demonstrated superior performance than traditional CNNs regarding rapid binary DPN classification. The transformer-based DLA offers a new direction for classifying DPN through automatic analysis of CCM images and holds potential for clinical screening.

## Data Availability

The raw data supporting the conclusions of this article will be made available by the authors, without undue reservation.
